# IgG4-related disease presenting as panuveitis without scleral involvement

**DOI:** 10.1186/s12348-017-0125-4

**Published:** 2017-02-27

**Authors:** Kinda Najem, Larissa Derzko-Dzulynsky, Edward A. Margolin

**Affiliations:** 1 Department of Ophthalmology, Université de Montréal, 5415 de l’Assomption Boulevard, Montreal, Quebec H1T 2M4 Canada; 2 Department of Ophthalmology, University of Toronto, 340 College Street Suite 501, Toronto, Ontario M5T 3A9 Canada; 3 801 Eglinton Avenue West, Suite 301, Toronto, Ontario M5N 1E3 Canada

**Keywords:** IgG4-related disease, Panuveitis, Cranial nerve palsies

## Abstract

**Background:**

The following case emphasizes the importance of including IgG4-related disease (RD) in the differential diagnosis of intraocular inflammation and multiple cranial nerve palsies.

**Results:**

A 33-year-old man, with a history of idiopathic bilateral panuveitis, presented with a new right pupillary-sparing partial third nerve palsy, which spontaneously resolved in 2 weeks, but was followed 1 month later, by a right sixth nerve palsy, which also resolved within a few weeks. Motility disturbance was accompanied by a decrease in the central acuity in the right eye. Magnetic resonance imaging/angiography (MRI/MRA) demonstrated a densely enhancing osteodestructive skull base process extending through the cavernous sinus and into the right superior orbital fissure. Biopsy of the lesion was consistent with IgG4-related disease (RD).

**Conclusions:**

This is the first reported case of IgG4-RD associated panuveitis without scleral involvement, expanding the list of clinical manifestations of the IgG4-RD.

## Review

A 33-year-old man, with a history of bilateral idiopathic panuveitis for the past 6 years, presented with a new onset of binocular oblique diplopia and right ptosis. He was previously treated with several immunomodulators, including cyclosporine, mycophenolate mofetil, azathioprine, and methotrexate. These agents had failed in controlling his active panuveitis, and he had been receiving adalimumab infusions every 2 weeks for the past 2 years.

On initial presentation, central acuity was 20/30 in each eye; extraocular motility testing demonstrated no adduction and severely restricted supraduction in the right eye, along with almost complete right-sided ptosis. Patient was diagnosed with a right pupillary-sparing partial third nerve palsy. Biomicroscopic examination revealed bilateral mild to moderate (1/2-1+) anterior uveitis with a few anterior vitreous cells but no posterior vitritis. Fundoscopy was grossly normal.

**Fig. 1:**
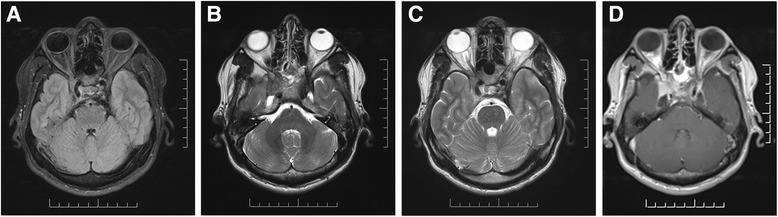
Axial T1 (**a**) and T2 (**b**, **c**) weighted magnetic resonance imaging, pre-gadolinium, showing an osteodestructive skull base process, with a lesion centered around the *right* sphenoid sinus and extending through the cavernous sinus into the adjacent meninges and *right* superior orbital fissure. Axial (**d**) T1 weighted magnetic resonance imaging, post-gadolinium, showing the focus of enhancement of the lesion

Urgent MRI and MRA of the brain and orbits were performed and revealed a densely enhancing osteodestructive skull base process, with a lesion centered around the right sphenoid sinus and extending through the cavernous sinus into the adjacent meninges and right superior orbital fissure (Fig. 1a–d). Radiological differential was between inflammatory (granulomatous non-infectious or infectious) or, less likely, neoplastic entities.

**Fig. 2:**
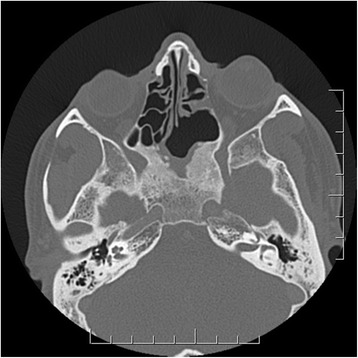
Axial computed tomography scanning of the brain demonstrating an asymmetric thickening in the *left* posterior cavernous sinus extending to the *right* anterior cavernous sinus and *right* orbital apex

When the patient was seen 2 weeks later, the right partial third nerve palsy had completely resolved. A computed tomography (CT) scanning of the brain and orbits was performed to further characterize the lesion and demonstrated a lack of normal inferior wall of the left sphenoid sinus with the small bone fragments in the sinus and a small tract extending from the left lateral sphenoid sinus wall towards the cavernous sinus. There was also an asymmetric thickening in the left posterior cavernous sinus extending to the right anterior cavernous sinus and right orbital apex (Fig. 2).

A month later, the patient started noticing diplopia again; there was complete absence of right abduction on exam compatible with a sixth nerve palsy. Central visual acuity in the right eye had deteriorated to counting fingers. There was a right RAPD, but no abnormalities on fundoscopy were noted.

CT scan of the chest, abdomen, and pelvis was obtained and was reported as normal. Multiple blood tests (complete blood count, erythrocyte sedimentation rate, C-reactive protein, antinuclear antibodies, syphilis screening, angiotensin-converting enzyme, and tuberculosis purified protein derivative skin testing) were all normal as well.

**Fig. 3:**
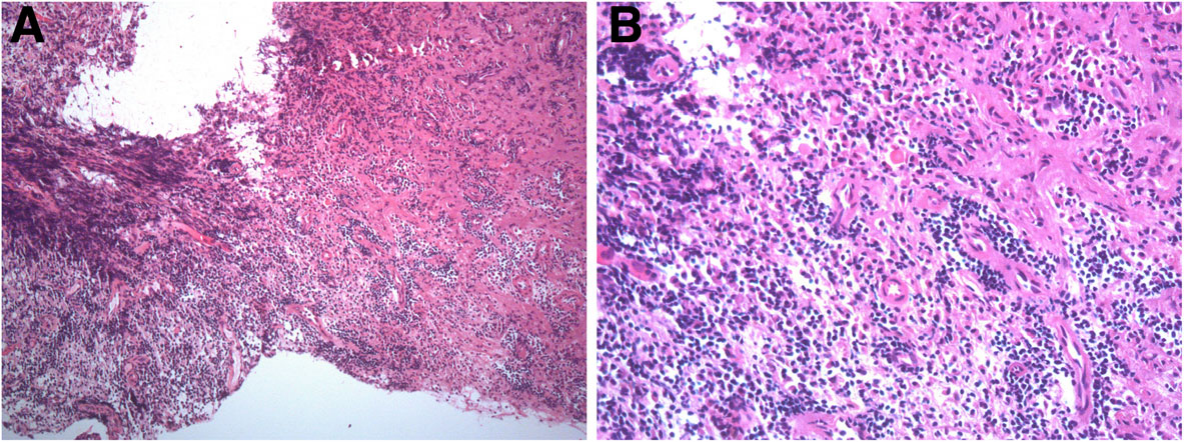
**a**, **b** Transsphenoidal biopsy of the skull base mass demonstrating an inflammatory infiltrate with lymphocytes and slight increase in B cells with interspersed macrophages and plasma cells

Transsphenoidal biopsy of the skull base mass was performed and demonstrated an inflammatory infiltrate with lymphocytes and slight increase in B cells with interspersed macrophages and plasma cells (Fig. 3a, b). Staining for CD20 and CD3 revealed a mixture of B and T cells. Histochemical staining for fungi and mycobacteria was negative. As the biopsy did not produce conclusive diagnostic results, a bone marrow biopsy and aspirate were performed to look for lymphomatous changes. Cytology and flow cytometry of the aspirate were both normal. Adalimumab was then discontinued as it was felt it could have been a possible culprit in the development of the skull base lesion (it has been previously reported to be associated with rare forms of lymphoma as well as increased incidence of sarcoidosis) [1–3].

One month after the biopsy was performed, visual acuity spontaneously improved to 20/40 on the right. Motility deficits had all resolved and fundoscopy remained normal. There was a mild anterior uveitis with a few anterior vitreous cells and no posterior vitritis in either eye.

**Fig. 4:**
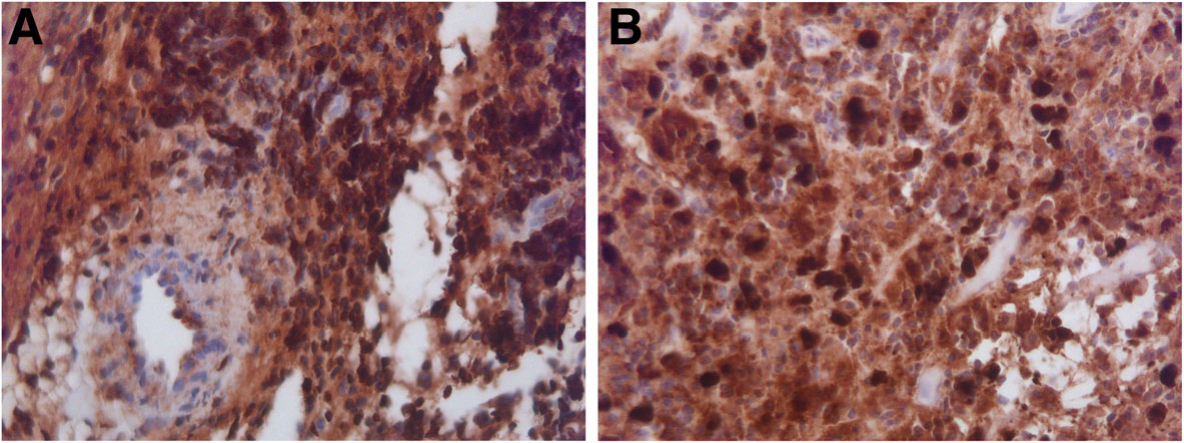
IgG stains (**a)** IgG4 stains (**b**) demonstrated an IgG:IgG4 ratio of more than 40% as well as more than thirty IgG4-positive plasma cells per high-power field

Lack of definitive diagnosis prompted repeat of the CT scan of the brain and orbits which was unchanged. At this point, a review of possible differential diagnosis led to another look at the results of the biopsy. IgG4 stains were requested and demonstrated an IgG:IgG4 ratio of more than 40% as well as more than 30 IgG4 positive plasma cells per high-power field (Fig. 4a, b). Serum IgG-4 levels were normal.

As the patient recovered spontaneously and has not had any future relapses, no treatment was initiated and he has been followed with observation only. The panuveitis has remained controlled with topical steroid drops over the past 2 years, after the initial presentation.

## Conclusions

To our knowledge, this is the first reported case of panuveitis without scleral involvement associated with IgG4-RD.

IgG4-RD is an emerging entity that has been described and reported on extensively in the recent years. It is characterized by tumefactive lesions, dense lymphoplasmacytic infiltrate abundant in IgG4-positive plasma cells, and fibrosis with typical storiform pattern and obliterative phlebitis, although the latter is only intermittently observed, depending on the affected organs [4, 5]. The disease was originally described in 2001 in a cohort of Japanese patients presenting with autoimmune pancreatitis and a raised serum IgG4 level [2, 3]. Since then, it has been described in nearly all organs, including the lungs, liver, kidney, and thyroid [4, 6–9].

Diagnostic criteria for IgG4-RD are still incompletely defined. An international consensus statement [10] had suggested three different levels of diagnostic classification for IgG4-RD: histologically highly suggestive of IgG4-RD, probable histologic features of IgG-RD, or insufficient histologic evidence of IgG4-RD. Other classifications [11] have attempted to include the serum IgG4 levels in the diagnostic criteria, but the serum levels can be normal in as much as 30–40% of patients (as they were in our case) with IgG4-RD thus rendering this diagnostic tool unsupportive in this cluster of patients [5, 12].

From an ophthalmological point of view, IgG4-RD most often affects the orbit or lacrimal gland [4, 9, 13]. A Japanese study reviewed 65 cases with IgG4-related ophthalmic diseases (ROD) [14] and reported 31 cases (57.7%) that had only lacrimal gland involvement, with the remaining 34 cases (52.3%) involving ocular adnexa: trigeminal nerve, extraocular muscles, orbit, eyelid and nasolacrimal duct [14]. Rare cases of conjunctival, scleral, and optic nerve involvement have also been reported [7, 15, 16].

Nearly half of the patients presenting with ocular IgG4-related ophthalmic disease will also have extraocular manifestations in other organs, and it is suggested that all patients should be evaluated for the salivary gland enlargement, lymphadenopathy, and lung, liver, and renal involvement [16]. Whole-body fluorodeoxyglucose positron emission tomography/computed tomography (FDG-PET/CT) is a valuable imaging study to assess for the extraocular manifestations of the disease [17].

In the past few years, associations between granulomatous diseases, such as sarcoidosis, adult orbital xanthogranuloma and granulomatosis with polyangiitis, and IgG4-RD have been described [15, 18–21]. These cases demonstrate a possible overlap between these two conditions, although this is still not clearly understood. Interestingly, lymphoma has been associated with IgG4-RD and the latter seems to increase the risk of lymphoma [17, 22–24]. Therefore, granulomatous disease and lymphoma should be considered in the differential of IgG4-RD. In the case presented here, both entities were initially considered but it was erroneously presumed that they were related to adalimumab use and not directly to IgG4-RD.

There is no clear consensus on the treatment of IgG4-RD. Spontaneous resolution has been described in the literature [25] and in our case, although the skull base lesion has remained unchanged, with the exception of mild panuveitis, the disease remained quiescent without treatment. Recurrence, however, is possible at any point in time. Corticosteroids are the first line of treatment and have been shown to be effective in treating the disease, although the relapse rate can be higher in patients treated with steroids [26]. Steroid-sparing agents have also been used successfully in treating IgG4 with rituximab (anti-CD20 monoclonal antibody) being the most successful. Dramatic responses to this anti-CD20 monoclonal antibody have been reported in the first month of treatment [27, 28]. Treatment with rituximab was suggested for our patient but, since he has remained largely asymptomatic, it was not used. Low-dose radiotherapy has also been described with variable response [17, 29].

Our report emphasizes the importance of keeping IgG4-RD in the list of differential diagnosis for patients with panuveitis and especially for patients presenting with panuveitis and eye motility abnormalities as these combinations of clinical findings are difficult to explain by a single pathological entity otherwise.
